# The Role of Releasing Incisions in Emergency Inguinal Hernia Repair

**DOI:** 10.3389/jaws.2023.11378

**Published:** 2023-06-07

**Authors:** Zachary N. Weitzner, David C. Chen

**Affiliations:** ^1^ Lichtenstein Amid Hernia Institute, University of California, Los Angeles, Los Angeles, CA, United States; ^2^ Department of Surgery, David Geffen School of Medicine, University of California, Los Angeles, Los Angeles, CA, United States

**Keywords:** inguinal hernia, hernia repair, robotic surgery, releasing incision, emergency hernia surgery

## Abstract

The majority of inguinal hernia repairs worldwide are performed on an outpatient basis. However, incarceration and concern for strangulation of abdominal contents necessitates emergent repair in order to address visceral ischemia. In the setting of salvageable ischemia, this necessitates release of strangulation of blood supply by the hernia defect and reduction of visceral contents into the abdominal cavity. In certain cases, this cannot be achieved with simple manual reduction, and requires enlargement of the aperture of the hernia defect with releasing incisions in order to allow reduction. We aim to describe strategies for releasing incisions via open, laparoscopic, and robotic approaches in emergency inguinal hernia repair.

## Introduction

Inguinal hernia repairs are one of the more common general surgical procedures performed worldwide, with estimates of greater than 20 million repairs performed annually worldwide and over 800,000 annually in the United States [1]. Studies have estimated approximately 9% of inguinal hernia repairs are performed emergently, most often because of incarceration, strangulation, and visceral compromise [2]. Emergent inguinal hernia repairs comprise significantly higher risk of morbidity and mortality compared to elective repair, up to 32% and 5%–5.5% compared to 8% and 0.2%–0.5% after elective repair, with the majority of risk due to visceral compromise due to strangulation [3–5]. In particular, these risks are elevated in individuals over 65 years of age, female patients, femoral hernias (especially right sided femoral hernias), those with prolonged symptom duration or multiple hernia-related hospitalizations in the year prior to presentation, bowel obstruction, and delay in treatment [3].

Inguinal hernias may be congenital or acquired. Regardless of cause, the principal of abdominal wall hernia formation is a defect in the musculo-aponeurotic wall allowing protrusion of subfascial contents through the defect, either from the peritoneum, pre-peritoneal space, or retroperitoneum. With advancements in cross-sectional imaging, exceedingly small hernia defects are being detected, with openings too small to allow herniation of structures. Similarly, hernia defects with exceptionally large apertures allow for free movement of structures. Hernia incarceration occurs when structures within the hernia sac are unable to be reduced back into their anatomical space, potentially leading to strangulation, when the blood flow to hernia structures becomes obstructed leading to ischemia. In defect apertures of intermediate size, structures within the hernia sac may be constricted at the level of the defect. This initially impedes the venous outflow resulting in edema and expansion of hernia structures, further preventing reduction of structures. Eventually, this edema leads to restriction of arterial inflow causing ischemia.

The mainstay of emergent hernia repair is to address the visceral compromise with reduction of hernia contents prior to the development of irreducible ischemia and subsequent repair of the hernia. It is important to recognize and prioritize in these circumstances, hernia is a secondary problem. Efforts to reverse visceral ischemia, prevent or control enteric spillage, and limit systemic sepsis are the priorities to limit morbidity and mortality associated with strangulated hernias. However, reduction of hernia contents, even operatively, is occasionally not possible due to the amount of visceral edema in the herniated structures resulting in a size mismatch between the herniated structures and hernia defect aperture. Additionally, strain on edematous, distended, and compromised bowel risks perforation and wound contamination, increasing the risk of morbidity. To allow safe reduction, releasing incisions may be required to enlarge the defect and reduce herniated viscera. This may be performed via an open approach, but can also be utilized in emergent minimally invasive laparoscopic and robotic hernia repairs. While releasing incisions have been described in operative lectures, anecdotes, and discussions, there is a paucity of literature describing their role in the practical management of emergency hernia surgery.

## Releasing Incisions in Open Surgery

The inguinal canal is a tubular structure comprised of four walls and two openings. The anterior wall is formed from the aponeurosis of the external and internal oblique muscles. Through the anterior wall, the superficial or external ring is formed in an opening of the anterior wall. This opening transitions to the covering of the inguinal contents. The deep ring, also known as the internal ring, is formed from the floor of the canal, which is comprised of the transversalis fascia and conjoint tendon. The roof of the canal is formed from the transversus abdominis, internal oblique, and part of the external oblique. The inferior wall of the canal is formed by the inguinal and lacunar ligaments [6].

In open inguinal hernia repair, the anterior wall is opened along the extent of the canal inferomedially to the external ring. Emergent repair involves reduction of dilated and strangulated viscera and reinforcement of the floor of the canal. Due to compression of venous outflow in strangulation, herniated visceral contents swell significantly after passing through the hernia defect, often making reduction difficult. In the majority of cases, application of circumferential pressure to squeeze edema out of the herniated viscera allows for ample size reduction to allow reduction of herniated contents through the hernia aperture. However, in emergency cases in which this fails and acute incarceration precipitates impending strangulation or perforation, the aperture size may be enlarged to allow for safe reduction of hernia contents.

**FIGURE 1:**
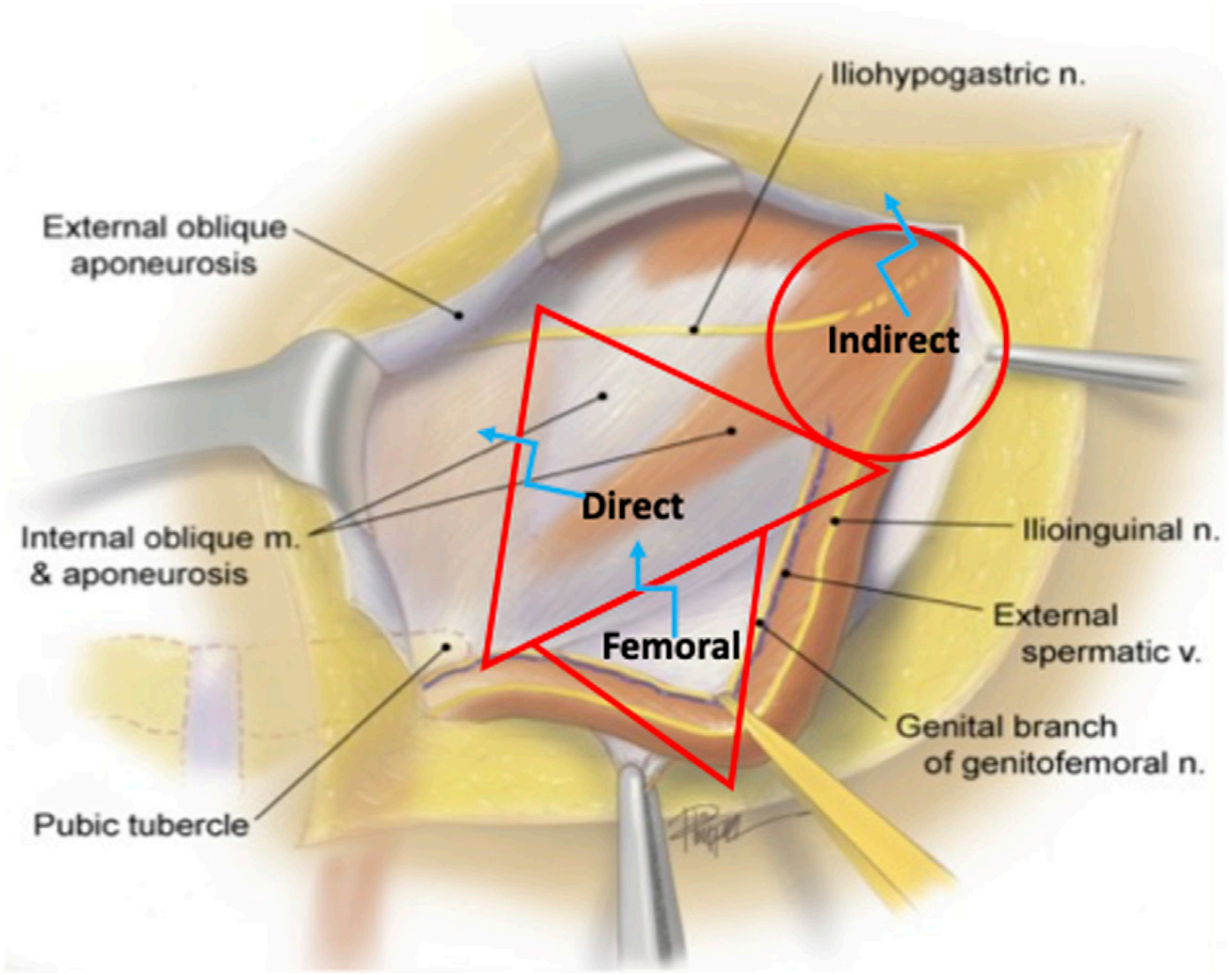
Open Inguinal Hernia Releasing Incisions. Indirect, direct, and femoral hernia spaces are outlined in red. The optimal sites for releasing incisions are marked with blue.

For indirect hernias, the viscera is herniated through the deep ring. Thus, when indirect hernia contents cannot be reduced manually through the deep ring, releasing incisions may be required to release the tension and allow for reduction of herniated viscera. In relation to the deep ring, the transversus abdominis marks the superior border, with the ilioinguinal nerve coursing posterior to it superolaterally. The inferior epigastric vessels mark the medial border of the deep ring, and the iliac vessels inferiorly. Thus, releasing incisions should be aimed cephalad and medially in the transversus abdominis muscle to avoid injury to the ilioinguinal nerve and inferior epigastric vessels. The iliohypogastric nerve typically courses cephalad and medial to the internal ring and can often be identified and avoided when opening the aperture of this orifice. In some cases, the iliohypogastric nerve may follow a subaponeurotic course running deep to this area, so releasing incisions should be made superficially in the fascial ring only and the extent minimized to limit potential transection (Figure 1).

Direct inguinal hernias pass through Hesselbach’s Triangle medial to the epigastric vessels in order to enter the inguinal canal. The boundaries of the direct defect are defined by the inguinal ligament inferolaterally, the deep ring and epigastric vessels superiorly, and the conjoined tendon and lateral border of the rectus medially. Opening the aperture of a direct defect in the cephalad direction risks bleeding from the epigastric vessels or injury to the spermatic cord. Inferolateral release in the inguinal ligament is unnecessarily destabilizing and risks neurovascular injury to the iliofemoral vessels, femoral nerve, anterior cutaneous nerve of the thigh, and femoral branch of the genitofemoral nerve. Thus, to minimize the risk of injury, releasing incisions made in the setting of a strangulated direct hernia should be made in inferomedially in the internal oblique or transversalis fascia directed toward the conjoined tendon and rectus abdominus muscle, as this is the safest border of the direct space for enlargement (Figure 1). The iliohypogastric nerve runs medial to the direct space coursing from the cephalad direction and care should be taken to identify and preserve this structure if possible.

**FIGURE 2:**
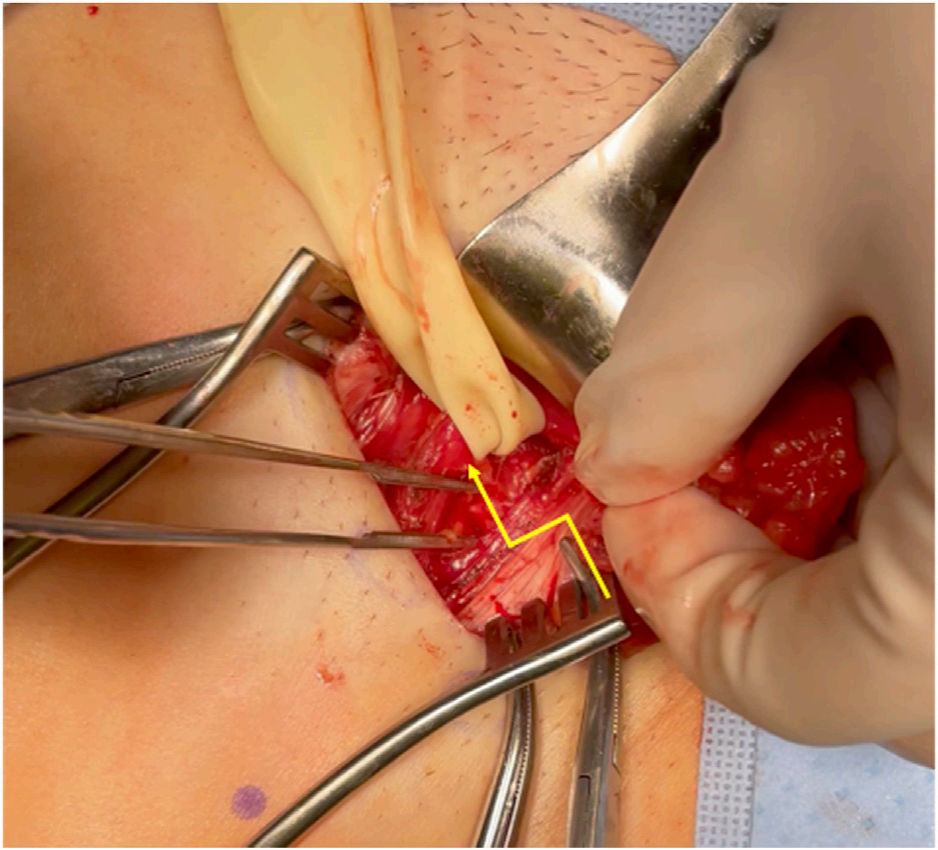
Open Femoral Hernia Releasing Incision. The yellow line marks the releasing incision of the inguinal ligament in femoral hernia repair.

Femoral hernia contents pass through the femoral canal inferior to the inguinal ligament, lateral to the lacunar ligament, above Cooper’s ligament, and medial to the femoral vessels. Thus, releasing incisions can safely be made by either opening the iliopubic tract if the floor of the inguinal canal is exposed, or the roof of the femoral canal, the inguinal ligament, if the thigh is exposed (Figures 1, 2). Incision towards the lateral aspect of the femoral canal risk damage to the femoral vessels, and medial incisions of Cooper’s ligament are inaccessible and ineffective. If division of the inguinal ligament is performed via and open approach, these should be repaired after visceral reduction, as they provide significant stability and anchoring of the anterior wall of the inguinal canal. In our practice, we reconstruct the released inguinal ligament with a permanent 2-0 Prolene suture.

## Releasing Incisions in Minimally Invasive Laparoscopic Surgery

**FIGURE 3:**
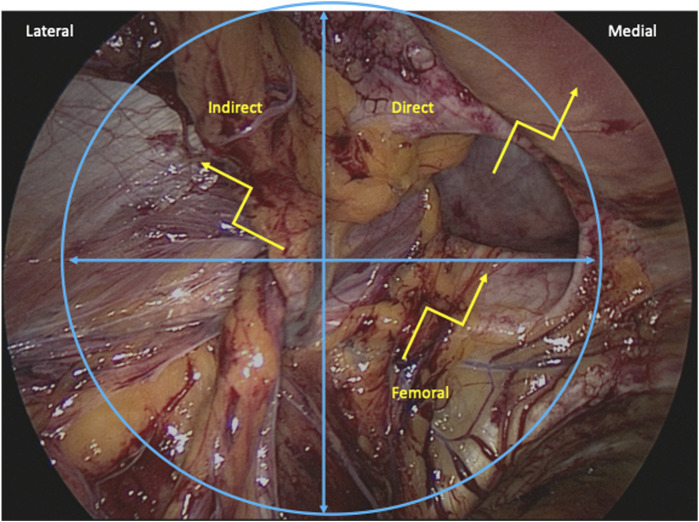
Minimally Invasive Inguinal Hernia Releasing Incisions. Indirect, direct, and femoral hernia spaces are outlined in blue. The optimal sites for releasing incisions are marked with yellow.

Traditionally, the majority of emergent hernia surgery for strangulation has been described via open approaches. However, as the proportion of surgeons trained to perform minimally inguinal hernia repairs increases, laparoscopy has been shown to be a safe approach for emergent inguinal hernia repair including in the context of acute incarceration and strangulation. This requires a comprehensive understanding of the posterior anatomy of the inguinal canal from a posterior view, described by Daes and Felix as the “critical view of the myopectineal orifice,” defined as the appropriate exposure of the anatomy of the posterior inguinal canal prior to mesh placement in laparoscopic and robotic inguinal hernia approaches [7]. From this view, the iliopubic tract divides the space into the suprainguinal and infrainguinal spaces, with direct and indirect inguinal hernias coursing through the suprainguinal space divided by the inferior epigastric vessels and femoral and obturator hernias in the infrainguinal space (Figure 3).

Indirect hernias are bound inferomedially by the epigastric vessels, inferolaterally by the iliopubic tract, and superiorly by the transversus abdominis and internal oblique muscle. Additionally, the spermatic cord traverses the internal ring from the inferior direction. To release strangulated indirect hernias from this posterior approach, releasing incisions should be made superolaterally in the transversus abdominis and internal oblique to avoid damage to the inferior epigastric vessels, cord, and neurovascular structures below the iliopubic tract. The genital nerve enters the inguinal canal from the inferolateral direction and is thus avoided. The extent of the releasing incision should be minimized to prevent inadvertent injury to the ilioinguinal nerve which runs superficial and superior to this space within the inguinal canal (Figure 3).

The direct space is bound inferolaterally by the iliopubic tract, superolaterally by the inferior epigastric vessels, and medially by the rectus abdominis. When releasing incisions are needed for direct hernias from this posterior approach, releasing incisions may be safely made towards the rectus abdominis in a superomedial direction, avoiding injury to the inferior epigastric and cord vessels that run laterally to this space (Figure 3). If incisions are made too deep, however, there may be risk to the cord structures as they pass through the inguinal canal anteriorly, so caution should be taken to pull towards the muscle and peritoneum during dissection. The extent of the releasing incision should be minimized to prevent inadvertent injury to the iliohypogastric nerve which runs superficial and superomedial to this space within the anterior inguinal canal.

Femoral hernias are bound superomedially by the iliopubic tract, medially by the lacunar ligament, superolaterally by the femoral vessels, and inferiorly by Cooper’s ligament. Releasing incisions should be made superomedially in the lacunar ligament or directly though the iliopubic tract which is seen from this view as the posterior aspect of the inguinal ligament. Releasing incisions in these approaches avoid damage to the iliac vessels. When mesh is placed in a posterior orientation from this approach, the iliopubic tract does not require reconstruction, in contrast to open femoral hernia releasing incisions, as the posterior placed mesh covering the myopectineal orifice provides support of the inguinal canal (Figure 3).

Obturator hernias are quite rare accounting for less than 1% of abdominal wall hernias, and are more common in thin elderly women, likely due to loss of supporting connective tissue and wider female pelvis. Incarceration and strangulation is occasionally encountered and poses a similar challenge. Understanding the boundaries of the obturator foramen can similarly direct a safe releasing incision in the setting of incarceration. The superolateral boundary of the obturator foramen heading in the direction of Cooper’s ligament is bound by the superior pubis ramus and division will not confer any significant release. An accessory obturator vein, referred to as the corona mortis, will often connect the iliac vein to the obturator vein and should be avoided. Posterolaterally, the obturator nerve, artery and vein will travel along the inner table of the pelvis and enter the obturator foramen. These neurovascular structures should be preserved and avoided. In the case of an incarcerated or strangulated obturator hernia, a releasing incision in the obturator internus muscle of the obturator membrane directed inferomedially heading directly down the pelvis away from Coopers and the neurovascular structures will allow for release and reduction of the contents of the obturator canal.

From a technical standpoint, when performing laparoscopic releasing incisions, we recommend using hook cautery with a pulling technique to direct cautery posteriorly, away from cord structures, neurovascular structures, and hernia contents. Alternatively, harmonic scalpel may be used with the hot blade oriented away from hernia contents in order to prevent inadvertent thermal injury (Supplementary Video S1). Monopolar shears are typically avoided or used only without energy to prevent secondary thermal injury to the entrapped viscera.

## Releasing Incisions in Robotic Surgery

Robotic approaches to emergent inguinal hernia repair are fundamentally the same as laparoscopic approaches, but with the distinct advantages of increased instrument articulation and enhanced optics and visualization. Use of robotic hook cautery allows for greater precision while making releasing incisions to allow incision of the aperture of the hernia neck by articulating the hook into the defect. Robotic shears may also accomplish similar maneuvers, and can be used without cautery or very focal energy depending on risk of thermal injury. Additionally, the availability of *in vivo* fluorescence imaging with indocyanine green (ICG) infusion provides an enhanced adjunct to assess visceral viability in these challenging cases.

In both robotic and laparoscopic approaches, the view of the myopectineal orifice allows intervention on incarcerated bowel prior to reduction in cases where irreversible ischemia has occurred prior to intervention. A vessel sealer may be used to devascularize the loop of compromised bowel, preventing systemic circulation of inflammatory cytokines after reducing the loop and relieving strangulation. Additionally, a stapler may be used to divide proximal and distal limbs of strangulated bowel prior to reduction to prevent spillage.

## Conclusion

Releasing incisions are beneficial in the technical management of incarceration and strangulation in emergent inguinal hernia management. A strong understanding of inguinal anatomy in both anterior and posterior approaches helps minimize potential collateral damage to both hernia contents and the native inguinal canal in order to minimize secondary risk and safely manage these challenging abdominal wall emergencies.

## Supplementary Material

SUPPLEMENTARY VIDEO S1 Laparoscopic Direct Inguinal Hernia Releasing Incision. Hook cautery is used to make a releasing incision in the rectus abdominis.
